# Unveiling new horizons in heart research: the promise of multi-chamber cardiac organoids

**DOI:** 10.1186/s13619-024-00193-y

**Published:** 2024-04-23

**Authors:** Junjie Hou, Ye-Guang Chen, Jing-Wei Xiong

**Affiliations:** 1 School of Basic Medical Sciences, Jiangxi Medical College, Nanchang University, Nanchang, 330031 China; 2 The State Key Laboratory of Membrane Biology, Tsinghua-Peking Center for Life Sciences, School of Life Sciences, Tsinghua University, Beijing, 100084 China; 3 Beijing Key Laboratory of Cardiometabolic Molecular Medicine, Institute of Molecular Medicine, College of Future Technology, and State Key Laboratory of Natural and Biomimetic Drugs, Peking University, Beijing, 100871 China

**Keywords:** Organ development, Cardiac organoid, Heart development

## Abstract

Human cardiac and other organoids have recently emerged as a groundbreaking tool for advancing our understanding the developmental biology of human organs. A recent paper from Sasha Mendjan’s laboratory published in the journal *Cell* on December 7, 2023, reported the generation of multi-chamber cardioids from human pluripotent stem cells, a transformative technology in the field of cardiology. In this short highlight paper, we summarize their findings. Their cardioids remarkably recapitulate the complexity of the human embryonic heart, including tissue architecture, cellular diversity, and functionality providing an excellent in vitro model for investigation of human heart development, disease modeling, precision medicine, and regenerative medicine. Thus, generating cardioids is an important step forward for understanding human heart development and developing potential therapies for heart diseases.

## Main text

In the realm of medical research, scientists are constantly pushing the boundaries of innovation to unlock a deeper understanding of complex organs and their associated diseases. Thanks to the revolutionary understanding of embryonic development and tissue homeostatic maintenance, scientists are taught to culture and replicate the architecture and function of human organs at a small scale in vitro, which are termed organoids (Lancaster and Knoblich 2014). These tiny, self-organized three-dimensional structures not only mimic the architecture, cellular composition, and some functions of an organ but also offer an unprecedented opportunity to reveal the intricacies of organ development, disease progression, and organ regeneration.

So far, the emerging organoids vary from brain organoids that mimic the complexity of the human brain's neural networks to gut organoids that replicate the structure and functionality of the intestines. Similarly, liver organoids offer insight into liver diseases and drug toxicity testing, while kidney organoids provide a unique model for understanding renal development and disease (Lancaster and Huch 2019). In spite of these advances in different organoids, cardiac organoids, which resemble both the structure and regular pacing waves and contractions of the heart, have only been reported in recent years (Hofbauer et al. 2021; Schmidt et al. 2023).

**Fig. 1:**
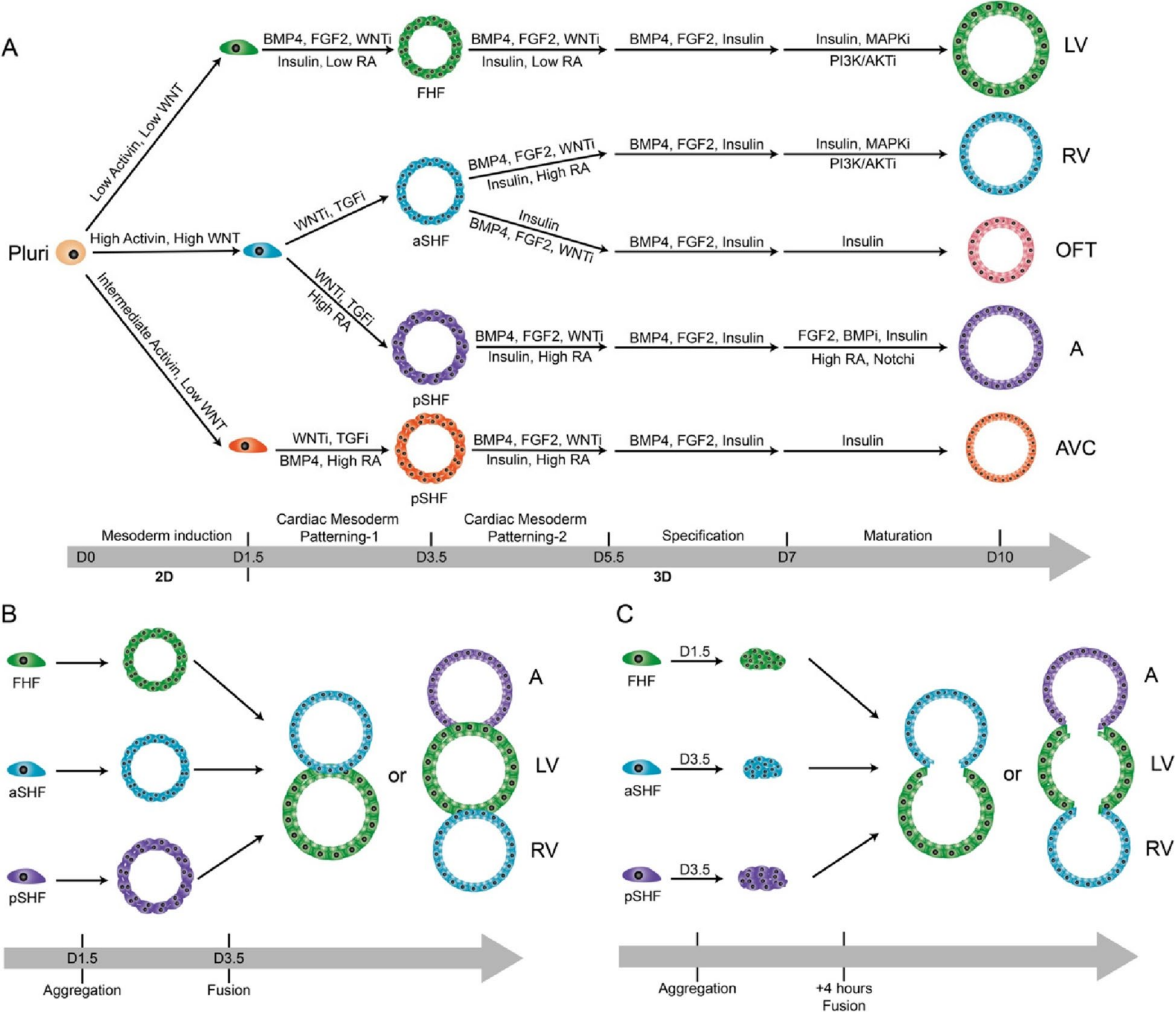
Schematic of experimental design in differentiation and fusion of cardioids. **A** Differentiation protocol of cardioids subtypes. **B** Experimental design of multi-chamber cardioid generation. **C** Experimental design of shared-lumen multi-chamber cardioid generation

Mendjan and colleagues recently established a human cardioid which contained the atrium, left ventricle (LV), right ventricle (RV), outflow tract (OFT), and atrioventricular canal (AVC) (Schmidt et al. 2023). This all-in-one cardioid recapitulates, for the first time, the structures of each heart chamber, mirrors cardiac contractions and calcium transients in vivo. Briefly, the authors described their remarkable experimental design, starting from culturing different subtypes of cardioids, and then fusing them together into a final multi-chamber cardioid. But generating these cardioids was not simple. During heart development, the first heart field (FHF) primarily gives rise to the LV, the anterior second heart field gives rise to the RV and most of the OFT, and the posterior second heart field gives rise to most of the atrium and a portion of the AVC. By temporally modulating several key cardiogenic signaling pathways such as ACTIVIN, bone morphogenic protein, fibroblast growth factor, retinoic acid, insulin, transforming growth factor-b, and WNT (wingless-related integration site), they first developed three main cardiac progenitor lineages and then differentiated them into five subtypes of cardioids that were enriched in mature cardiomyocytes (Fig. 1A). Transcriptomic analysis of these cardioids revealed gene signatures that corresponded to the LV (IRX3, IRX4, HEY2 and MYL2), RV (ISL1, IRX1, HEY2 and RFTN1), atrium (TBX5, NR2F2 and NR2F1), OFT (WNT5A, ISL1, HAND2 and RSPO3), and AVC (TBX2 and TBX3). In the further maturation and functional specification progress, the LV/RV cardioids elevated ventricular structure protein MYL2, chamber marker NPPA and NPPB, and the MYH7/MYH6 ratio resulting in paralleled sarcomere structures and higher contraction amplitude. Moreover, the OFT cardioids displayed more efficient smooth muscle cell (SMC) differentiation propensity and AVC cardioids comprised a few PECAM^+^ or COL1A1^+^ cells. These physical traits accurately reflected functional capabilities. And in their 3D differentiation protocol, they particularly noted that precise cell counting before patterning-1 aggregation was essential for robust cardioid formation since a higher cell density always led to inefficient cardiomyocyte differentiation and neural marker expression within the organoid core.

To determine the similarity of cellular and molecular characteristics of these cardioids to human cardiogenesis in vivo, the authors carried out single-cell RNA sequencing (scRNA-seq) of these cardioids and made a comparative analysis with published scRNA-seq datasets from human embryonic hearts. They found remarkable similarities of cardioids to human ventricular and atrial cardiomyocytes. Functionally, cardioid automaticity had a greater extent of contractions in the LV, atrium and AVC, while the RV and OFT had a weaker capacity to spontaneously contract. Moreover, this automaticity decreased in the LV, RV and OFT cardioids by a loss of HCN4 K^+^/Na^+^ channel expression. In addition, Ca^++^ transients and action potentials of each cardioid revealed a distinct identity. The LV cardioids had a prolonged Ca^++^ transient and a more homogeneous signal propagation field potential spread compared with the atrial and AVC cardioids as reported in vivo. Patch-clamp analysis of single cardiomyocytes revealed that the action potential duration (APD) in the atrial/AVC cardioids was shorter than in the RV cardiomyocytes, which was consistent with primary human cardiomyocytes. Overall, the fetal-like electrochemical signaling of cardioids recapitulates well the human heart biology.

To generate a multi-chamber cardioid, the authors applied both cardiac progenitor sorting and co-developing cardioid strategies (Fig. 1B). The former strategy enabled the self-sorting of different cardiomyocytes but did not result in a multi-chamber cardioid. By co-developing cardioids in the latter strategy, they were able to generate an all-in-one cardioid, which achieved structural connections and maintained distinct identities and compartments of each cardioid. Interestingly, they noted that cardioids co-develop only when combined at day 3.5, were electrochemically coupled, and contracted in a coordinated manner by day 6.5. Remarkably, if they substituted all the cavity structures with respective cardiac progenitors on the second day of culture (LV at day 1.5; RV at day 3.5; atrium at day 3.5), they surprisingly generated cardioids with a shared lumen (Fig. 1C). The Ca^++^ propagation in these cardioids was not unidirectional at the beginning, but it became gradually dominated from the atria in pacing after 3 days in culturing. Thus, this multi-chamber cardioid provides a great platform for mimicking human heart development, disease modeling, and drug screening. For example, they generated cardioids from each of the mutants ISL1, TBX5 or FOXF1 hiPSCs, and found similar cardiac chamber-specific defects as those in clinical congenital heart diseases. In addition, they observed the expected effects of ion channel drugs on either single or multi-chamber cardioids. These results made this multi-chamber cardioid a powerful tool when combined with genome editing to dissect cellular mechanisms of congenital heart diseases and carry out high-throughput drug screening for clinical translations. Together, these data suggest that cardioids generated in this work faithfully recapitulate human heart structures and physiology, which serve well for studying both basic cardiogenesis research and clinical translation in the future.

## Conclusions

This work reports the first multi-chamber cardioids with fetal-like identity of cardiomyocytes and have distinct features of cardiomyocytes from different chambers. These cardioids have beating waves and unidirectional Ca^++^ transients initiated from the atria as in the normal human heart. Their molecular markers and ion channel genes of cardiomyocytes in these cardioids have striking similarity to human cardiogenesis in vivo. Therefore, this cardioid offers a credible human development platform for genome editing, disease modeling, therapeutic drug screening, and regenerative medicine. While having these remarkable advances, current protocols for generating cardioids are quite complex and warrant further investigations, particularly from different laboratories. Cell-type diversity and advanced cardiac modeling such as trabeculation, septation, circulation, ballooning, and coronary vasculature are the next milestones to achieve. Nevertheless, like other organoids, generating cardioids in this work represents a great in vitro model for understanding both human heart development and improving cardiovascular healthcare in the future.

## Data Availability

Not applicable.
